# Cardiovascular risk evaluation and antiretroviral therapy effects in an HIV cohort: implications for clinical management: the CREATE 1 study

**DOI:** 10.1111/j.1742-1241.2010.02424.x

**Published:** 2010-08

**Authors:** M Aboud, A Elgalib, L Pomeroy, G Panayiotakopoulos, E Skopelitis, R Kulasegaram, C Dimian, F C Lampe, A Duncan, A S Wierzbicki, B S Peters

**Affiliations:** 1 HIV Medicine, Guy’s & St Thomas’ HospitalsLondon, UK; 2 Sexual Health/HIV, Beckenham HospitalLondon, UK; 3 Royal Free Hospital, University College LondonLondon, UK; 4 HIV & STDs, King’s College LondonLondon, UK

## Abstract

**Aims:**

The aim of this study is to determine the cardiovascular disease (CVD) risk profile of a large UK HIV cohort and how highly active antiretroviral therapy (HAART) affects this.

**Methods:**

It is a cross-sectional study within a large inner city hospital and neighbouring district hospital. A total of 1021 HIV positive outpatients representative of the complete cohort and 990 who had no previous CVD were included in CVD risk analysis. We recorded demographics, HAART history and CVD risk factors. CVD and coronary heart disease (CHD) risks were calculated using the Framingham (1991) algorithm adjusted for family history.

**Results:**

The non-CVD cohort (*n* = 990) was 74% men, 51% Caucasian and 73.1% were on HAART. Mean age was 41 ± 9 years, systolic blood pressure 120 ± 14 mmHg, total cholesterol 4.70 ± 1.05mmol/l, high-density lipoprotein-C 1.32 ± 0.48 mmol/l and 37% smoked. Median CVD risk was 4 (0–56) % in men and 1.4 (0–37) % in women; CHD risks were 3.5 (0–36) % and 0.6 (0–16) %. CVD risk was > 20% in 6% of men and 1% of women and > 10% in 12% of men and 4% of women. CVD risk was higher in Caucasians than other ethnicities; the risk factor contributing most was raised cholesterol. For patients on their first HAART, increased CHD risk (26.2% vs. 6.5%; odds ratio 4.03, p < 0.001) was strongly related to the duration of therapy.

**Conclusions:**

Modifiable risk factors, especially cholesterol, and also duration of HAART, were key determinants of CVD risk.

**Discussion:**

Regular CHD and/or CVD risk assessment should be performed on patients with HIV, especially during HAART therapy. The effect of different HAART regimens on CHD risk should be considered when selecting therapy.

**What’s known**

There is a higher incidence of observed cardiovascular disease in HIV patients, and this is increased with HIV therapy. Much of the increase has been ascribed to smoking within patients with high risk lifestyle, and the contribution of therapy and its duration to increasing cholesterol is uncertain.

**What’s new**

This study demonstrates that cholesterol, rather than smoking, is the most important contributor to increased predicted CV/coronary heart disease risk (CHD) risk in our HIV cohort. It also shows that duration of HAART is key, and hence has important implications for the screening and management of patients with HIV infection.

## Introduction

Anti-retroviral therapy (HAART) has reduced traditional HIV-associated disease and death (1). Cardiovascular disease (CVD) has emerged as a major cause of morbidity and mortality in HIV following initial reports of dyslipidaemia (2–4), probably as a result of the combination of the pro-inflammatory effects of HIV infection, an increased prevalence of traditional risk factors (RFs) and the effects of HAART (5–8).

Cardiovascular disease risk screening is an increasing priority in national health care strategies (9). Risk is calculated using tools derived from epidemiological studies including the USA Framingham study (10), UK primary care databases (QRISK) (11) and European prospective cohort studies (SCORE) (12). In all these calculators, CVD or CHD risk is calculated using age, gender, smoking, systolic blood pressure (SBP) and total cholesterol: high-density lipoprotein cholesterol (TC : HDL-C) ratio (13). *Ad hoc* adjustments can be made to CVD risk factor profiles to add the effects of family history of premature CHD, ethnicity and obesity (13). HIV cohorts in the UK, and most of the developed world, are an ‘ageing population’, and hence there is an increasing need to focus on CVD. Traditional CVD RFs are increased in HIV populations (14,15) and seem to predict risk in a similar fashion to uninfected populations (16).

This study was designed to describe the CVD RF burden in a large HIV cohort and to apply these findings to recommendations for clinical practice.

## Methods

### Setting

Cardiovascular risk evaluation and antiretroviral therapy (CREATE) is a cross-sectional study looking at estimated 10-year CVD and CHD risk, and the metabolic syndrome in HIV, and this study, CREATE 1, concentrates on the former aspects. Recruitment was from a large inner London teaching hospital with a large HIV cohort (exceeding 2500) and a medium-sized local district hospital with a more affluent demographic.

### Study population

Patients at both hospitals are from a diverse ethnic and socioeconomic group. The study recruited from June 2005 to September 2006.The protocol was approved by the St Thomas’ Hospital Ethics Committee. To minimise selection bias, all HIV-infected outpatients were eligible providing they were regular attendees for their general HIV care and were not pregnant. Patients were not recruited from specialist clinics, such as the lipodystrophy or hepatitis co-infection clinics, to avoid unfair weighting of patients with metabolic issues. These patients were still open to study entry from their general HIV clinic attendance. The research staff identified clinics for inclusion, and all such patients were offered entry.

### Data collection

A structured proforma was used to collect data on demographics, basic anthropometry, HIV and HAART history, current CD4 cell counts, HIV viral loads and details of co-infections. Ethnicity was self reported and categorised as: Afro-Caribbean, black African, white (Caucasian), Asian and other. Details of HAART were recorded, including the date of commencement, protease inhibitor use and the current regimen.

Anthropometric, physiological and biochemical parameters required to define cardiovascular risk (CVR) and the metabolic syndrome were collected. The use of anti-atheroma therapies, including antithrombotic, antihypertensive, lipid-lowering and hypoglycaemic drugs, was collected, and the use of recreational drugs recorded. Weight, height, waist circumference and blood pressure were measured by trained experienced research nurses or doctors using standard criteria. Blood pressure was measured in the sitting position after 5 min rest with an Omron 320 (Henfield, West Sussex, UK) automated system calibrated to British Hypertension Society standards. Blood lipids were measured fasting when possible, and assayed on a Roche Hitachi (Lewes. Sussex, UK) platform by standard techniques of CHOD-PAP for cholesterol and a non-ionic precipitation method for HDL-cholesterol. Data were entered directly onto the proforma during clinic attendance. Missing information on the database was checked with the source data, clinical notes, on scheduled review. The database was regularly cross-checked with the source data.

The estimated risk of CVD and CHD was calculated using the Framingham (1991) (10) equation recommended for UK use by both the Joint British Societies Guidelines (2005) (17) and the National Institute for Health and Clinical Excellence (NICE) guidelines (2008) (18). No adjustment was made for ethnicity as the population was of diverse origin and no specific correction factors exist for West Africans. CHD risk relates to the development of coronary heart disease (MI, CHD death, angina, coronary insufficiency). CVD additionally includes stroke, congestive heart failure and peripheral vascular disease.

To assess CVD risk in a control population, data were compared with the QRESEARCH database (19), which is derived from a UK general practice population, a self-referred CVD risk programme in the UK (HEART-UK/Unilever CVD risk assessment study) (20) and with the combined DAD cohort studies of CVR in HIV (15,21) (Table 1A–C). The DAD study (data collection on adverse events of anti-HIV drugs) is a collaboration of eleven prospective cohorts of HIV-infected individuals from across Europe, Australia and the US, totalling over 30,000 participants.

**Table 1:** (A–C) Clinical characteristics of male and female patients attending a HIV service in the UK compared with a general cardiovascular risk screening group in the UK and the DAD cohort study

	**Cohort without CVD (male; *n* = 737)**	**HEART-UK (male; *n* = 27,776)**
**(A) HIV positive men in CREATE 1 compared with a HEART-UK population**		
Age	41.2 ± 9.2	51.5 ± 16.2*
Caucasian (%)	65	Not available
Smoking (%)	45*	13.4
Systolic blood pressure (mmHg)	121 ± 14	140 ± 17*
Hypertension (%)	12	13
Total cholesterol (mmol/l)	4.70 ± 1.05	5.10 ± 1.00*
HDL-cholesterol (mmol/l)	1.25 ± 0.44	1.20 ± 0.40
Diabetes (%)	2	4
CHD (+) (%)	NA	11
BMI (kg/m^2^)	24.6 ± 3.8	-
Waist > 102 cm (%)	24	25

	**Cohort without CVD (female; *n* = 253)**	**HEART-UK (female; *n* = 43,261)**
**(B) HIV-positive women in CREATE 1 compared with a HEART-UK population**		
Age	38.8 ± 8.8	52.1 ± 15.4*
Caucasian (%)	9	Not available
Smoking (%)	16*	12.7
Systolic blood pressure (mmHg)	118 ± 14	134 ± 19*
Hypertension (%)	9	13
Total cholesterol (mmol/l)	4.74 ± 1.04	5.20 ± 1.00*
HDL-cholesterol (mmol/l)	1.54 ± 0.54	1.50 ± 0.40
Diabetes (%)	2	3
IHD (+) (%)	NA	8.7*
BMI (kg/m^2^)	27.8 ± 6.1	-
Waist > 88 cm (%)	64*	40.1

	**Complete CREATE 1 cohort, including CVD (*n* = 1022)**	**DAD (*n* = 23,468)**
**(C) Complete cohort in CREATE 1 compared with subjects in the DAD study**		
Age	40 (35–46)	39 (34–47)
Caucasian (%)	51	–
Male (%)	76	75
MSM	44	45
Smoking (%)	37*	51
Systolic blood pressure (mmHg)	120 (110–130)	120 (110–130)
Hypertension (%)	12*	8.5
Total cholesterol (mmol/l)	4.7 (3.9–5.3)	5.0 (4.2–6.0)
HDL-cholesterol (mmol/l)	1.23 (1.00–1.53)	1.1 (0.9–1.4)
Diabetes (%)	3	2.5
IHD (+) (%)	3	1.4
CD4 count (mm^3^/l)	406 (289–562)	418 (255–612)
Viral Load (log)	< 1.70 (< 1.70–3.54)	< 2.7 (< 2.7–6.9)
BMI (kg/m^2^)	24.7 (22.3–27.7)	23 (21–25)
Waist > 102 cm (%)	24	–
Family history early CHD (%)	13	11.7

IHD, ischaemic heart disease, CREATE 1, cardiovascular risk evaluation and antiretroviral therapy; HDL, high-density lipoprotein; CHD, coronary heart disease; BMI, body mass index; MSM, men who have sex with men.

### Statistical analysis

Statistical analysis was performed with spss (Cheltenham, Gloucestershire, UK). This analysis included only patients free of pre-existing CHD diagnoses. Estimated 10-year CHD/CVD risk was summarised using medians and the proportion with values > 10% and > 20% per decade. Subgroups were compared using Chi-squared or Fishers exact test for categorical variables and Mann–Whitney tests for continuous variables. Logistic regression was used to assess the association of HAART use with 10-year CHD risk > 10% and to investigate the extent to which this association was independent of traditional CHD risk factors. The association of duration of HAART use with 10-year CHD risk was examined in a subgroup of patients on first line HAART.

## Results

### Patient characteristics

A total of 1021 patients were recruited, of which 990 were free of pre-existing CVD (Table 1). For the purposes of data presentation and analysis (Table 1A,B), the total numbers in CREATE1 were 990 and exclude patients with established CVD. For purposes of comparison with the DAD study cohorts, which included CVD, data from the complete cohort of 1021 were used (Table 1C).

The majority, 74%, were men, 50.8% were white, 6.9% Afro-Caribbean, 33.3% African (various), 1.9% Asian and 6.9% from other ethnic groups. Men who have sex with men (MSM) comprised 43.9%, and 41% were heterosexual. Intravenous drug abuse was reported by 1.65%. The median HIV viral load was 50 copies/ml and CD4 count 406 cells/mm^3^, and 73.1% were on HAART. CVD was present in 3% of both men and women, and diabetes in 3% and 2%, respectively. The mean (±SD) age was 41 ± 9 years (48.6%≥ 40 years). Current or recent smoking (within 5 years) was reported by 37% and was commoner in whites (51% vs. 23%; p = 0.02) than black Africans. The SBP was 120 ± 14 mmHg, and 11% had a SBP > 140 mmHg. The TC was 4.70 ± 1.05 mmol/l and HDL-C was 1.32 ± 0.48 mmol/l. An elevated TC (> 5 mmol/l) was present in 35.6%. HDL-C < 1 mmol/l in men or < 1.2 mmol/l in women was found in 33%. An elevated TC : HDL ratio > 5 was present in 18.7% and > 6 in 7.6%. Lipid-lowering drugs had been prescribed in 10.8%.

The male population in CREATE 1 was younger (41 years vs. 52 years), contained more smokers (45% vs.13%), had lower SBP (121 mmHg vs. 140 mmHg), slightly lower TC (4.7 mmol/l vs. 5.1 mmol/l) and less established CHD than those self-referring for CVD risk screening in the HEART-UK/Unilever study. Similarly, women recruited to CREATE 1 were younger (39 years vs. 52 years), had lower SBP (118 mmHg vs. 134 mmHg) and cholesterol (4.7 mmol/l vs. 5.2 mmol/l) and less CHD (3% vs. 9%), but more smoked (16% vs.13%). HEART-UK/Unilever participants were markedly different in ethnic origin (92% were non-Caucasian) and were more centrally obese (64% vs. 40%). The population was similar to that recruited to the DAD study cohorts (Table 1C) except for marginally lower TC (4.7 mmol/l vs. 5.0 mmol/l), higher HDL-C (1.23 mmol/l vs. 1.1 mmol/l) and body mass index (BMI) (24.7 kg/m^2^ vs. 23 kg/m^2^) and a lower prevalence of current smoking (37% vs. 51%).

### Cardiovascular and CHD

The median CVD risk was 4 (0–56) %/decade in men and 1.4 (0–37) %/decade in women, whereas CHD risks were 3.5 (0–36) %/decade and 0.6 (0–16) %/decade, respectively. CVD risk was > 20% in 6% of men and 1% of women and > 10% in 12% of men and 4% of women, whereas CHD risk exceeded 10% in 13% of men and 2% of women and was > 20% in 2% of men but no women. CVD risk was lower in all decade cohorts than the HEART-UK/Unilever study, although our patients were significantly younger and had a higher prevalence of smoking (Table 1A,B). There was suggestion of increased CHD risk among men in our cohort compared with general population seen by general practitioners (19). In this study for men aged 45–55 years, CHD risk > 15% was present in 14.7% compared with 7.76% in the control group. There were insufficient data to make meaningful comparisons for women.

Analyses were performed to investigate the associations of gender, ethnicity (Caucasians vs. non-Caucasians), risk group (MSM vs. heterosexuals) and use of HAART with CVD and CHD risk. The median CVD and CHD risks were 4.4% and 2% for men and 1.4% and 1% for women (p < 0.001). The proportions with CVD risk > 10% were 21.8% vs. 3.8% (p < 0.001) and for CHD risk ≥ 10% 16.4% vs. 0.4% (p < 0.001), respectively. Statistically significant differences in CHD risk were also seen for those in older age groups, and groups stratified by TC above or below 5 mmol/l, SBP above or below 140 mmHg and smokers vs. non-smokers (data not shown).

Compared with non-Caucasians, Caucasians had greater CVD and CHD risks, and approximately a 3-fold increase in the prevalence of CHD risk > 10% or CVD risk > 20%. These differences could partly be attributed to more men among our Caucasians (96% vs. 53%; p < 0.001) hence more MSM individuals (73% vs. 13%; p < 0.001), higher viral load (2.63 vs. 2.33; p < 0.001), but also higher median CD4 count (432 vs. 381; p < 0.001), more smoking (50% vs. 22%; p < 0.001) and lower HDL-C (1.22 vs. 1.42 mmol/l; p < 0.001). No significant differences in CVD or CHD risk were observed in MSM compared with male heterosexuals, despite significant differences in age (40 vs. 42 years; p < 0.001), smoking (50% vs. 37%; p < 0.001) and prevalence of drug-treated hypertension (5% vs. 11%; p = 0.003) and higher viral loads (2.66 vs. 2.33; p < 0.001) and CD4 counts (434 vs. 381; p < 0.001).

### HAART use

A total of 705 patients out of 973 were on HAART; 245 were on their first line regimen, of whom 44.1% had used HAART for < 1 year, and about a third and a quarter used HAART for 1–3 years and > 3 years, respectively.

Patients on HAART had significantly raised median CVD risk (3.57% vs. 2.34%; p = 0.01) and CHD (0.64% vs. 0.38%; p < 0.001) risk, associated with increased age (42 vs. 37 years, p < 0.001), higher TC (4.86 vs. 4.24 mmol/l; p < 0.001), but also lower viral load (1.96 vs. 4.02; p < 0.001) and higher HDL-C (1.39 vs. 1.12 mmol/l; p < 0.001) (Table 2).

**Table 2:** Association of factors with current HAART use

***n/N* (%)**	**Using HAART (*n* = 705)**	**Not using HAART (*n* = 258)**	**p value (Chi-squared/Fishers exact test)**
Male	509/705 (72.2)	204/258 (79.1)	0.031
Age > 40 years	388/705 (55.0)	83/258 (32.2)	< 0.001
Caucasian	330/690 (47.8)	149/253 (58.9)	0.003
MSM	279/705 (39.6)	144/258 (55.8)	< 0.001
Chol > 5 mmol/l	296/705 (42.0)	47/258 (18.2)	< 0.001
Lipid-lowering drug use	88/705 (12.5)	6/258 (2.3)	< 0.001
HDL < 1 mmol/l	134/705 (19.0)	95/258 (36.8)	< 0.001
TC/HDL ratio > 4.5	192/705 (27.2)	88/258 (34.1)	0.037
Sys BP > 140 mmHg	77/705 (10.9)	29/258 (11.2)	0.89
Smoker	250/705 (35.5)	107/258 (41.5)	0.087

HAART, highly active antiretroviral therapy; MSM, men who have sex with men; HDL, high-density lipoprotein; TC, total cholesterol; Sys BP, systolic blood pressure.

The duration of HAART was associated with CVD and CHD risk. For this analysis, only the 245 participants on first line therapy were included to avoid confounding from previous HAART use. The proportions with 10 year CVD and CHD risk > 20% for those on HAART < 1 year were 4.3% and 0.8% and for ≥ 3 years exposure 11% and 4.8%, respectively (p = 0.01). These differences were statistically significant for both Caucasians and non-Caucasians. A further analysis was performed to define variables within this group associated with increased CVD risk with duration of HAART. Age ≥ 40 years, TC ≥ 5 mmol/l and SBP≥ 140 mmHg were all more common in the group who had been on HAART the longest. Conversely, there were fewer persons with low HDL-C in the ≥ 3 year compared with < 1 year HAART groups, and the TC/HDL ratio did not vary with length of time on HAART (Table 3B). Smoking was not a factor that contributed to a higher CHD risk for those on HAART for longer periods (as a group), as there were similar proportions of smokers in each group regardless of the duration on HAART.

**Table 3:** (A, B) Mean lipid values according to HAART use

**HAART now**		**Total cholesterol**	**HDL-cholesterol**	**TC : HDL ratio**
**(A) Mean total cholesterol, HDL-cholesterol and total/HDL ratio by current HAART use**				
No	Mean	4.28	1.14	4.05
	*N*	258	258	258
	SD	0.89	0.34	1.32
Yes	Mean	4.88	1.40	3.84
	*N*	705	705	705
	SD	1.06	0.51	1.34
Total	Mean	4.72	1.33	3.90
	*N*	963	963	963
	SD	1.05	0.49	1.34

**Years 1st line**		**Total cholesterol**	**HDL-cholesterol**	**TC : HDL ratio**
**(B) Mean total cholesterol, HDL-cholesterol and total/HDL ratio by years on first line**				
< 1	Mean	4.50	1.23	3.98
	*N*	108	108	108
	SD	0.97	0.42	1.27
1–3	Mean	4.81	1.41	3.75
	*N*	76	76	76
	SD	0.96	0.50	1.33
≥ 3	Mean	5.31	1.49	3.91
	*N*	61	61	61
	SD	0.86	0.49	1.39
Total	Mean	4.80	1.35	3.89
	*N*	245	245	245
	SD	0.99	0.48	1.32

HDL, high-density lipoprotein; HAART, highly active antiretroviral therapy; TC, total cholesterol.

The contribution that each Framingham equation variable made to estimated CVD/CHD risk in patients on HAART was investigated. The unadjusted odds ratio for high CVD risk was 1.36 (0.92, 2.00) for HAART users vs. non-users. Adjusting for age and gender, the odds ratio fell to 1.13 (0.72, 1.80). Similarly, the unadjusted odds ratio for high CHD risk was 1.59 (0.99, 2.57) for HAART users vs. non-users and after adjustment for age and gender, the odds ratio fell to 1.02 (0.57, 1.85). This excess risk was accounted for after adjustment for SBP, smoking and TC; the biggest fall in odds ratio was observed after correction for cholesterol alone (0.63 (0.34–1.20).

**Figure 1:**
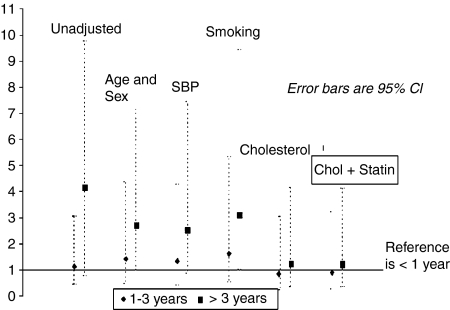
Comparison of coronary heart disease (CHD) risk with length of treatment with highly active antiretroviral therapy (HAART) before and after adjustment for CHD risk factors. Contribution of CV risk factors to odds ratio (OR) for 10 year CHD risk > 10% by length of time on HAART. *n* = 243 first line HAART users only (OR *Y*-axis). First adjustment for age/gender; then sequentially for sBP, smoking and total Chol. The degree of OR drop reflects the contribution of that factor to risk in the first place. Biggest OR drop seen when TC corrected for

A similar analysis was performed for 245 patients on first-time HAART according to the duration of HAART (< 1 year, 1–3 years, ≥ 3 years) (Figure 1). The unadjusted odds ratio for high CHD risk was 1.24 for 1–3 years and 5.13 for ≥ 3 years groups, compared with the < 1 year group (Figure 1). Again the greatest contribution to the excess risk in both the 1–3 years and ≥ 3 years groups was TC, as indicated by the comparatively large fall in odds ratio after adjustment for TC.

## Discussion

The increased frequency of observed CHD in HIV-infected vs. uninfected patients has focused attention on appropriate strategies to prevent cardiovascular disease in this population. Observational cohort studies have shown an increase in observed CVD events with the use of HAART, particularly with protease inhibitors (15,21). Some of the protease inhibitor effect can be attributed to drug-associated dyslipidaemia (21).

Among our UK-based cohort in CREATE1, levels of CVD and CHD risk appeared high compared with similar age groups in the general population, with a higher prevalence of smoking and hypercholesterolaemia. The average CVD and CHD risks in our cohort were 6.0% and 4.15%, respectively. The CHD risk was much lower than the 7–7.4% CHD risk seen in the Italian SIMONE cohort of HIV-infected patients (22,23), where the prevalence of elevated CVD risk > 10% was 17% and CHD risk> 10% was 10%. CVD risk > 20% was present in 4.9% and CHD risk > 20% in 1.6%. Other cohort studies of HIV-infected patients have shown higher prevalence of CHD risk > 10% of 23% and > 20% in 8% (24) or 11% (14). In the DAD study, the same version of the Framingham risk scoring tool used in our study determined that the CHD risk was 2.4% (25). Thus, the population recruited to CREATE 1 is at the intermediate risk for CVD compared with other HIV cohort studies. The CHD risk in the CREATE 1 cohort was elevated compared with a similar cohort of the general population from the QRESEARCH database, and the proportions with elevated CVD risk were similar to sex-matched age cohorts from the self-selected general population recruited for the HEART-UK/Unilever Study (20).

We found in CREATE 1 that caucasians were much more likely to have a CVD or CHD risk ≥ 10% than non-Caucasians. Even amongst the non-Caucasians, the proportion with raised CVD or CHD risk increased with the duration of HAART (from 3.5% with < 1 year use to 17.9% with > 3 years use; *n* = 245 first line users only). While many guidelines recommend adjustment of the Framingham risk for additional risk factors, this remains controversial. The Framingham risk calculator was derived from data on a mainly white working class population and predicted the correct proportion at risk in patients with HIV in DAD cohort study (25). Aside from Caucasians, the largest ethnic sub-group in CREATE 1 was African and diverse with respect to country of origin. Secondary adjustment for ethnicity is possible and tends to reduce CVD and CHD risk in African-derived populations compared with Asian or Caucasian populations in the UK (26), but is limited and is mostly based on West Africans. The degree of adjustment required for other African populations has not been determined, but CVD risk is elevated in urban or migrant populations (27,28). In this study as CVD and CHD risks had already been adjusted for family history, further adjustment was not performed (17). In Afro-Caribbean general population, the age-adjusted prevalence of CVD is 0.61, but there are no data on patients with HIV with this ethnic background in the UK.

In our study, the increased predicted CVD and CHD risk among Caucasians is at variance with a large prospective HIV cohort study of observed acute myocardial infarctions from the USA (29), where African-American race was a significant predictor of acute myocardial infarction, with a relative risk 1.43 compared with non-African-Americans. This may well reflect the differences in underlying risk factors between the 2 cohorts, such as proportions of smokers, differences in BMI and dysglycaemia and effects of social deprivation. Similar discrepancies were found between our cohort and the same US cohort in respect of gender, as among our female patients there were very few with even a moderately high predicted CVD or CHD risk. There was a marked increase in the relative rate of women with observed acute myocardial infarctions in the same US HIV cohort, with a relative risk of 2.98, compared with the control population (29).

In this study, smoking was less frequent than in the DAD cohort (37% vs. 52%) and showed pronounced ethnic and gender differences. Smoking was most associated with CVD risk in men and was commoner in Caucasians. In women, the prevalence of smoking was slightly increased compared with the general population. A large proportion of the cohort was heterosexual black African, where smoking was less prevalent than among Caucasians (23% vs. 51%). Therefore, the relative contribution of smoking to CVD risk in this population is likely to be lower than in other studies.

The greatest population-attributable risk in the INTERHEART study was dyslipidaemia. Dyslipidaemia is frequent in HIV (25). Dyslipidaemia related to the duration of HAART therapy contributed most to an increased predicted CVD and CHD risk amongst our cohort. The duration of first line HAART use was associated with a CHD risk ≥ 10%, with a relative risk > 5 for those on HAART for over 3 years compared with those who on HAART for under 1 year. This finding replicates those of the DAD study recruited from 1999 to 2003 (21) and shows that despite the availability of newer protease inhibitors that do not significantly affect lipid concentrations, most patients were still receiving HAART regimens that induced dyslipidaemia and increased CHD risk.

These findings provide a rationale for specific policies for the management of CVR in people with HIV, using adaptations of standard guidelines from the Joint British Societies (17) or NICE (18). This study demonstrates that management of CVD risk in HIV patients should consider length of exposure to HAART as well as ethnicity and gender. Risk calculators give only broad estimates of risk (30) and additional risk stratification may be required in intermediate (10–20%) risk patients (31,32). Patients at 10–20% risk should be prioritised for intensive lifestyle interventions. Modifiable risk factors should be actively managed, including the use of antihypertensives and lipid-lowering agents where lifestyle changes such as diet and exercise do not suffice. It is important for the individual and the patient cohort to reduce rates of smoking, and to attain optimum body weight (5,6). If these intervention strategies are not successful in reducing CHD risk, then changing HAART regimens should be considered (33–35). The observation that duration of first line HAART correlated strongly with CHD risk has clinical implications for management. It is important that CHD is assessed before and after treatment, to ascertain the extent to which the HAART might be contributing to increased CHD risk. There are a number of antiretroviral agents that do not affect lipids to the extent of other current therapies. These include the newer generation protease inhibitors, such as atazanavir (36), although this advantage is mitigated in part by the concomitant use of ritonavir as a pharmacological enhancer (37). Other agents that have a favourable lipid profile include raltegravir, an integrase inhibitor (38), as well as some of the agents that have been available for many years, such as nevirapine (34,35).

## Abbreviations

CREATE1: Cardiovascular Risk Evaluation and Antiretroviral Therapy Effects (Study name)
BMI: body mass index
CHD: coronary heart disease risk
CVD: cardiovascular disease risk
CVR: cardiovascular risk
HDL: high-density lipoprotein
IHD: ischaemic heart disease
MSM: men who have sex with men
(s)BP: (systolic) blood pressure
TC: total cholesterol

## Supporting information

Data S1. Algorithm for management of HIV-associated CVR/lipodystrophy.

## References

[1] Bozzette SA, Ake CF, Tam HK (2003). Cardiovascular and cerebrovascular events in patients treated for human immunodeficiency virus infection. N Engl J Med.

[2] Braitstein P, Yip B, Heath KV (2003). Interventional cardiovascular procedures among HIV-infected individuals on antiretroviral therapy 1995–2000. AIDS.

[3] Dube MP, Sprecher D, Henry WK (2000). Preliminary guidelines for the evaluation and management of dyslipidemia in adults infected with human immunodeficiency virus and receiving antiretroviral therapy: recommendations of the Adult AIDS Clinical Trial Group Cardiovascular Disease Focus Group. Clin Infect Dis.

[4] Currier JS (2002). Cardiovascular risk associated with HIV therapy. J Acquir Immune Defic Syndr.

[5] Kulasegaram R, Peters BS, Wierzbicki AS (2005). Dyslipidaemia and cardiovascular risk in HIV infection. Curr Med Res Opin.

[6] Aboud M, Elgalib A, Kulasegaram R (2007). Insulin resistance and HIV infection: a review. Int J Clin Pract.

[7] Wierzbicki AS, Purdon SD, Hardman TC (2008). HIV lipodystrophy and its metabolic consequences: implications for clinical practice. Curr Med Res Opin.

[8] Currier JS, Lundgren JD, Carr A (2008). Epidemiological evidence for cardiovascular disease in HIV-infected patients and relationship to highly active antiretroviral therapy. Circulation.

[9] Vascular Programme (2008). Putting Prevention First – Vascular Checks: Risk Assessment and Management.

[10] Anderson KM, Odell PM, Wilson PW (1991). Cardiovascular disease risk profiles. Am Heart J.

[11] Hippisley-Cox J, Coupland C, Vinogradova Y (2008). Predicting cardiovascular risk in England and Wales: prospective derivation and validation of QRISK2. BMJ.

[12] Conroy RM, Pyorala K, Fitzgerald AP (2003). Estimation of ten-year risk of fatal cardiovascular disease in Europe: the SCORE project. Eur Heart J.

[13] Wierzbicki AS, Reynolds TM (2009). Vascular risk screening: possible or too much, too soon?. Int J Clin Pract.

[14] Bergersen BM, Sandvik L, Bruun JN (2004). Elevated Framingham risk score in HIV-positive patients on highly active antiretroviral therapy: results from a Norwegian study of 721 subjects. Eur J Clin Microbiol Infect Dis.

[15] Friis-Moller N, Weber R, Reiss P (2003). Cardiovascular disease risk factors in HIV patients – association with antiretroviral therapy. Results from the DAD study. AIDS.

[16] Law MG, Friis-Moller N, El-Sadr WM (2006). The use of the Framingham equation to predict myocardial infarctions in HIV-infected patients: comparison with observed events in the D:A:D Study. HIV Med.

[17] British Cardiac Society, British Hypertension Society, Diabetes UK (2005). JBS 2: the Joint British Societies’ guidelines for prevention of cardiovascular disease in clinical practice. Heart.

[18] National Institute for Health and Clinical Excellence (2008). Lipid Modification.

[19] EMIS National User Group (2009). University of Nottingham. QResearch Database. QResearch Group. http://www.qresearch.org.

[20] Neil HA, Perera R, Armitage JM (2008). Estimated 10-year cardiovascular risk in a British population: results of a national screening project. Int J Clin Pract.

[21] Friis-Moller N, Sabin CA, Weber R (2003). Combination antiretroviral therapy and the risk of myocardial infarction. N Engl J Med.

[22] De Socio GV, Martinelli L, Morosi S (2007). Is estimated cardiovascular risk higher in HIV-infected patients than in the general population?. Scand J Infect Dis.

[23] De Socio GV, Parruti G, Quirino T (2008). Identifying HIV patients with an unfavorable cardiovascular risk profile in the clinical practice: results from the SIMONE study. J Infect.

[24] Knobel H, Jerico C, Montero M (2007). Global cardiovascular risk in patients with HIV infection: concordance and differences in estimates according to three risk equations (Framingham, SCORE, and PROCAM). AIDS Patient Care STDS.

[25] Law M, Friis-Moller N, Weber R (2003). Modelling the 3-year risk of myocardial infarction among participants in the Data Collection on Adverse Events of Anti-HIV Drugs (DAD) study. HIV Med.

[26] Brindle P, May M, Gill P (2006). Primary prevention of cardiovascular disease: a web-based risk score for seven British black and minority ethnic groups. Heart.

[27] Steyn K, Levitt NS, Hoffman M (2004). The global cardiovascular diseases risk pattern in a peri-urban working-class community in South Africa. The Mamre study. Ethn Dis.

[28] Cappuccio FP, Oakeshott P, Strazzullo P (2002). Application of Framingham risk estimates to ethnic minorities in United Kingdom and implications for primary prevention of heart disease in general practice: cross sectional population based study. BMJ.

[29] Triant VA, Lee H, Hadigan C (2007). Increased acute myocardial infarction rates and cardiovascular risk factors among patients with human immunodeficiency virus disease. J Clin Endocrinol Metab.

[30] Reynolds TM, Twomey P, Wierzbicki AS (2002). Accuracy of cardiovascular risk estimation in patients without diabetes. J Cardiovasc Risk.

[31] Hsue PY, Lo JC, Franklin A (2004). Progression of atherosclerosis as assessed by carotid intima-media thickness in patients with HIV infection. Circulation.

[32] de Saint ML, Vandhuick O, Guillo P (2006). Premature atherosclerosis in HIV positive patients and cumulated time of exposure to antiretroviral therapy (SHIVA study). Atherosclerosis.

[33] Currier JS, Lundgren JD (2008). Guidelines for managing cardiovascular risk: an evolving area. Curr Opin HIV AIDS.

[34] Carr A, Hudson J, Chuah J (2001). HIV protease inhibitor substitution in patients with lipodystrophy: a randomized, controlled, open-label, multicentre study. AIDS.

[35] Barragan P, Fisac C, Podzamczer D (2006). Switching strategies to improve lipid profile and morphologic changes. AIDS Rev.

[36] Colafigli M, Di GS, Bracciale L (2008). Cardiovascular risk score change in HIV-1-infected patients switched to an atazanavir-based combination antiretroviral regimen. HIV Med.

[37] Bentue-Ferrer D, Arvieux C, Tribut O (2009). Clinical pharmacology, efficacy and safety of atazanavir: a review. Expert Opin Drug Metab Toxicol.

[38] Markowitz M, Nguyen BY, Gotuzzo E (2007). Rapid and durable antiretroviral effect of the HIV-1 integrase inhibitor raltegravir as part of combination therapy in treatment-naive patients with HIV-1 infection: results of a 48-week controlled study. J Acquir Immune Defic Syndr.

