# Supplementary material for: Cardiovascular risk evaluation and antiretroviral therapy effects in an HIV cohort: implications for clinical management: the CREATE 1 study
Source: Int J Clin Pract. 2010 Aug;64(9):1252–9. doi: 10.1111/j.1742-1241.2010.02424.x (PMC2913108; doi:10.1111/j.1742-1241.2010.02424.x)
Supplement: Supplementary file 1 [file ijcp0064-1252-SD1.ppt]

## Slide 1
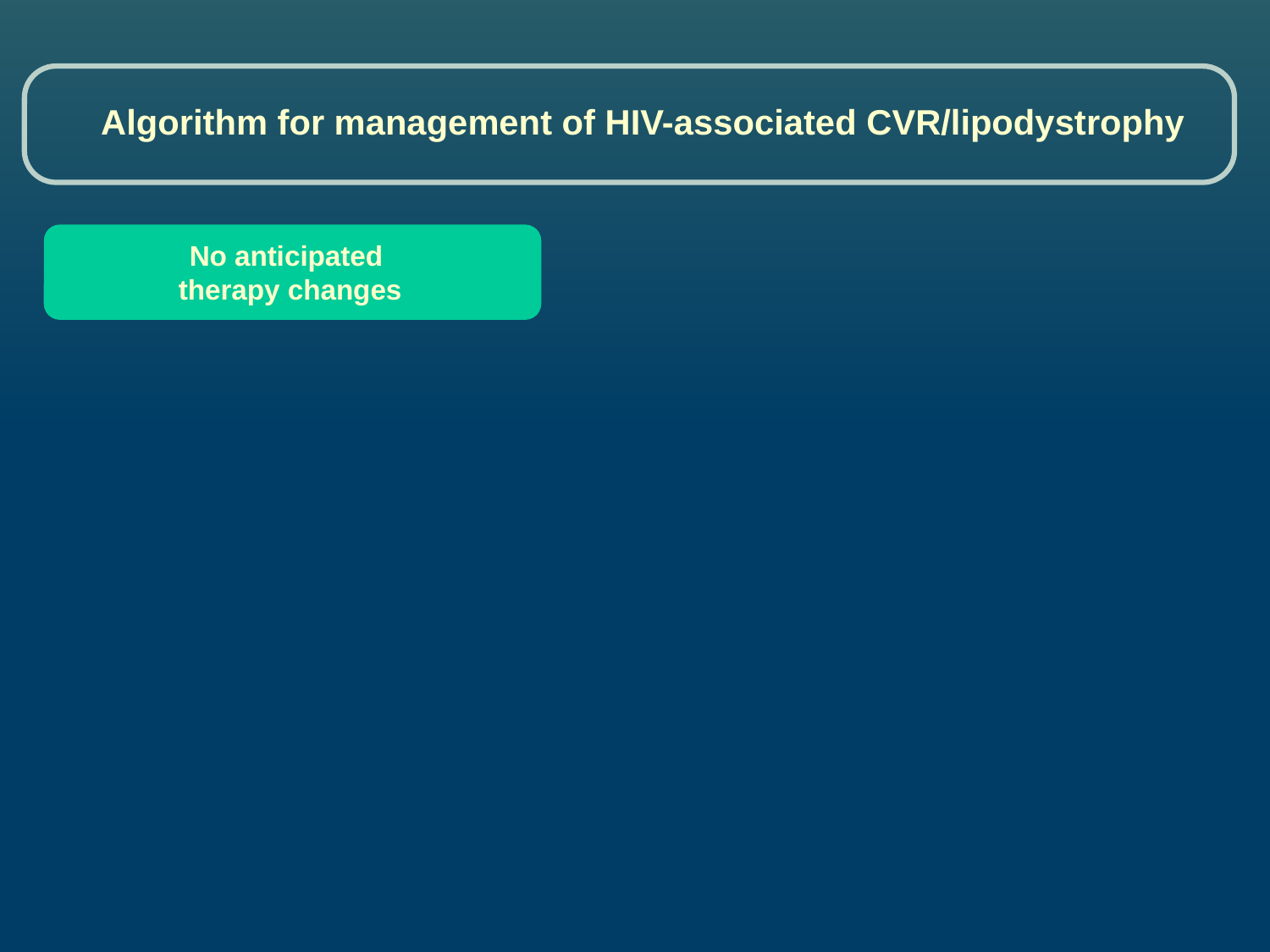

Algorithm for management of HIV-associated CVR/lipodystrophy
No anticipated
therapy changes

## Slide 2
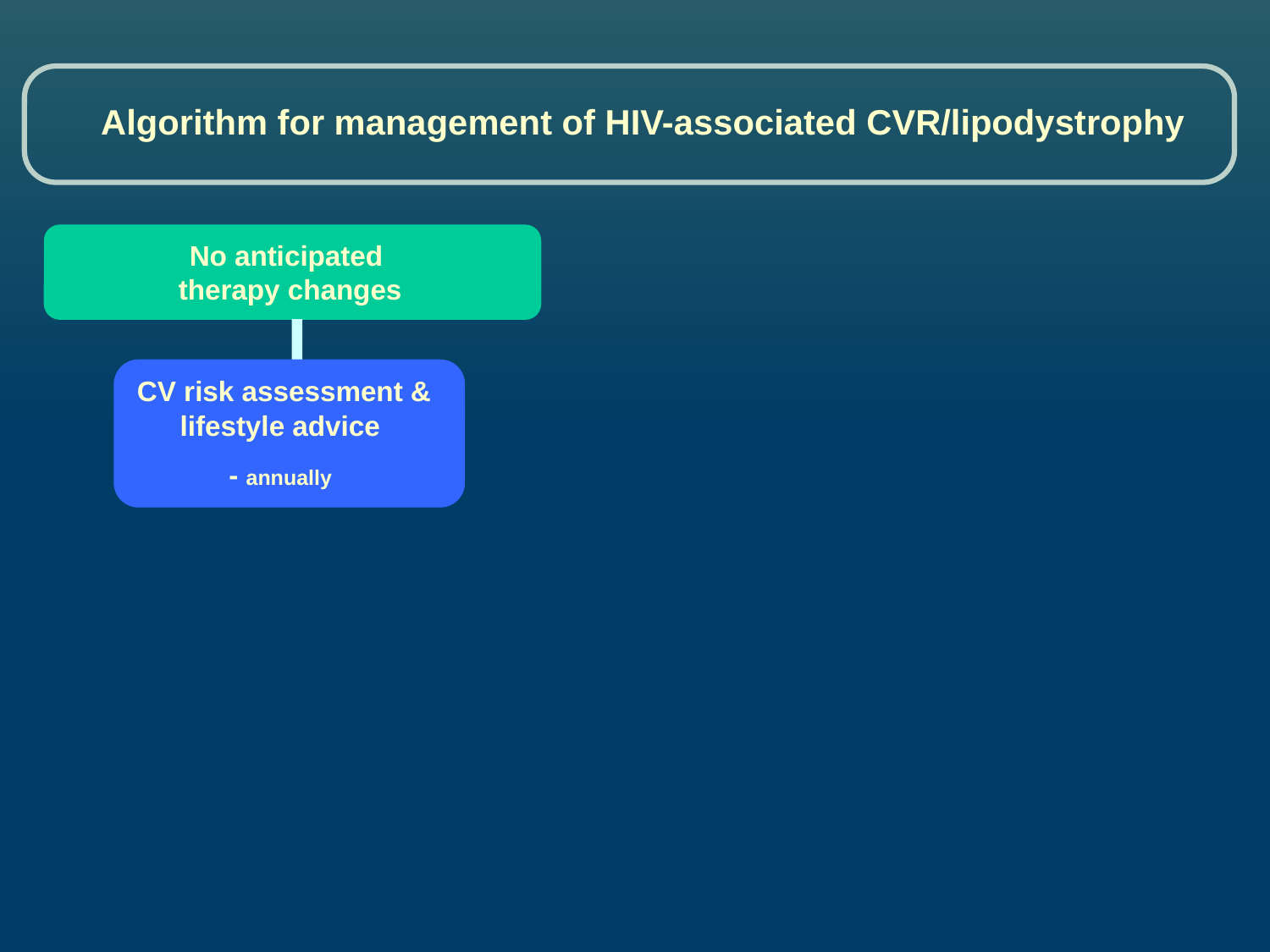

Algorithm for management of HIV-associated CVR/lipodystrophy
No anticipated
therapy changes
CV risk assessment & lifestyle advice
- annually

## Slide 3
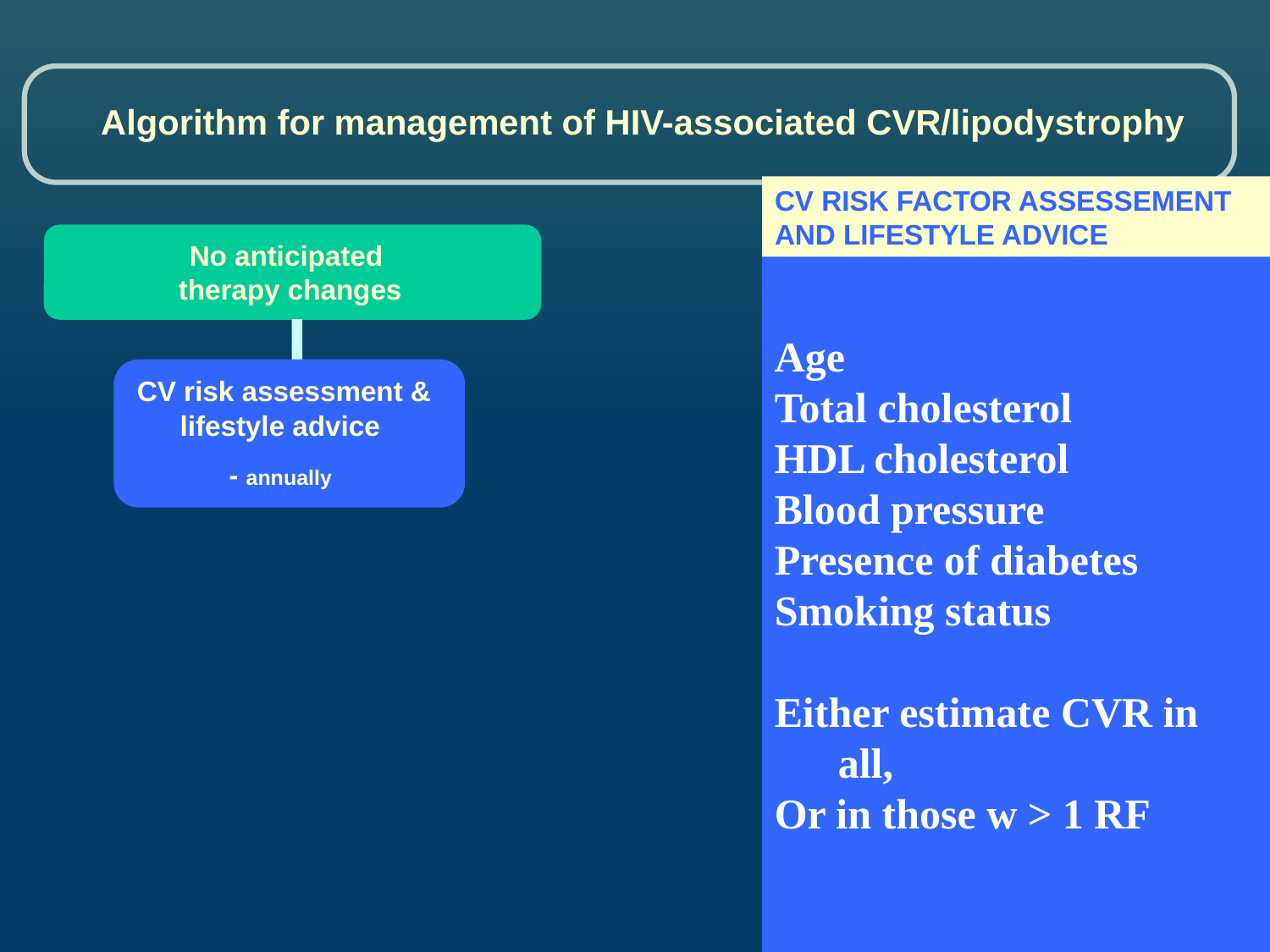

Algorithm for management of HIV-associated CVR/lipodystrophy
CV RISK FACTOR ASSESSEMENT AND LIFESTYLE ADVICE
No anticipated
therapy changes
Age
Total cholesterol
HDL cholesterol
Blood pressure
Presence of diabetes
Smoking status
Either estimate CVR in all,
Or in those w > 1 RF
CV risk assessment & lifestyle advice
- annually

## Slide 4
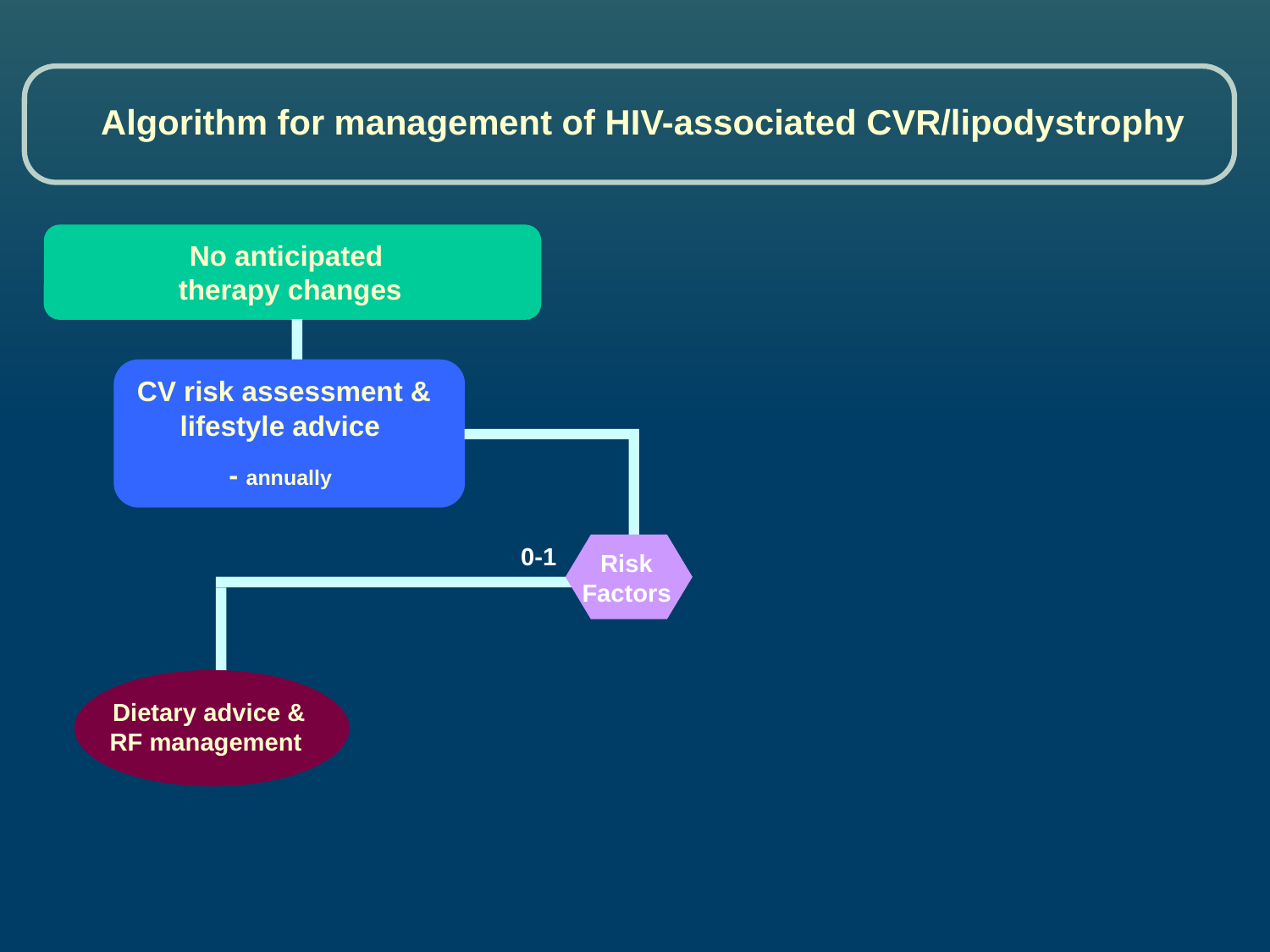

Algorithm for management of HIV-associated CVR/lipodystrophy
No anticipated
therapy changes
CV risk assessment & lifestyle advice
- annually
0-1
Risk Factors
Dietary advice &
RF management

## Slide 5
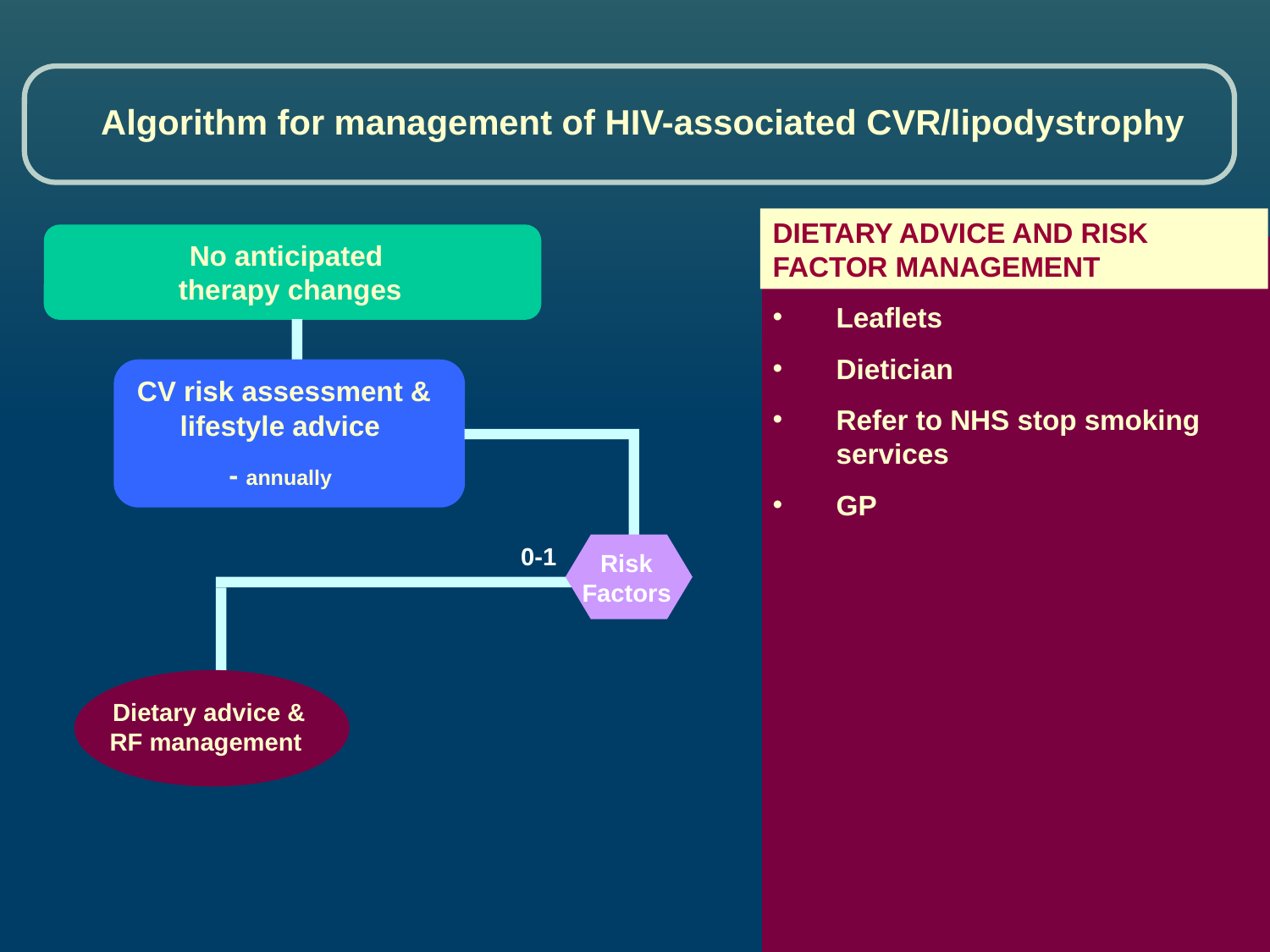

Algorithm for management of HIV-associated CVR/lipodystrophy
DIETARY ADVICE AND RISK FACTOR MANAGEMENT
No anticipated
therapy changes
Leaflets
Dietician
Refer to NHS stop smoking services
GP
CV risk assessment & lifestyle advice
- annually
0-1
Risk Factors
Dietary advice &
RF management

## Slide 6
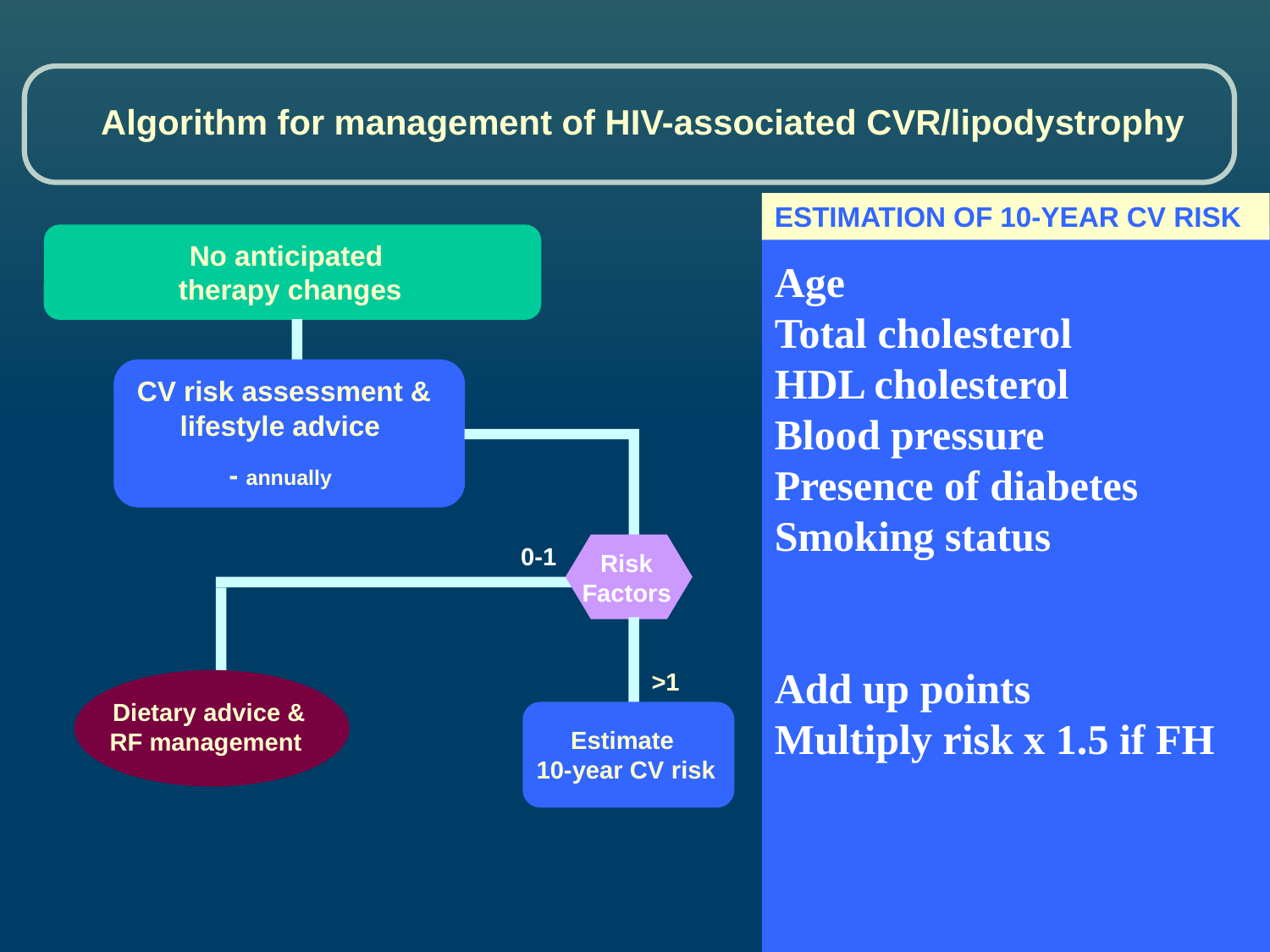

Algorithm for management of HIV-associated CVR/lipodystrophy
ESTIMATION OF 10-YEAR CV RISK
No anticipated
therapy changes
Age
Total cholesterol
HDL cholesterol
Blood pressure
Presence of diabetes
Smoking status
Add up points
Multiply risk x 1.5 if FH
CV risk assessment & lifestyle advice
- annually
0-1
Risk Factors
Dietary advice &
RF management
>1
Estimate
10-year CV risk

## Slide 7
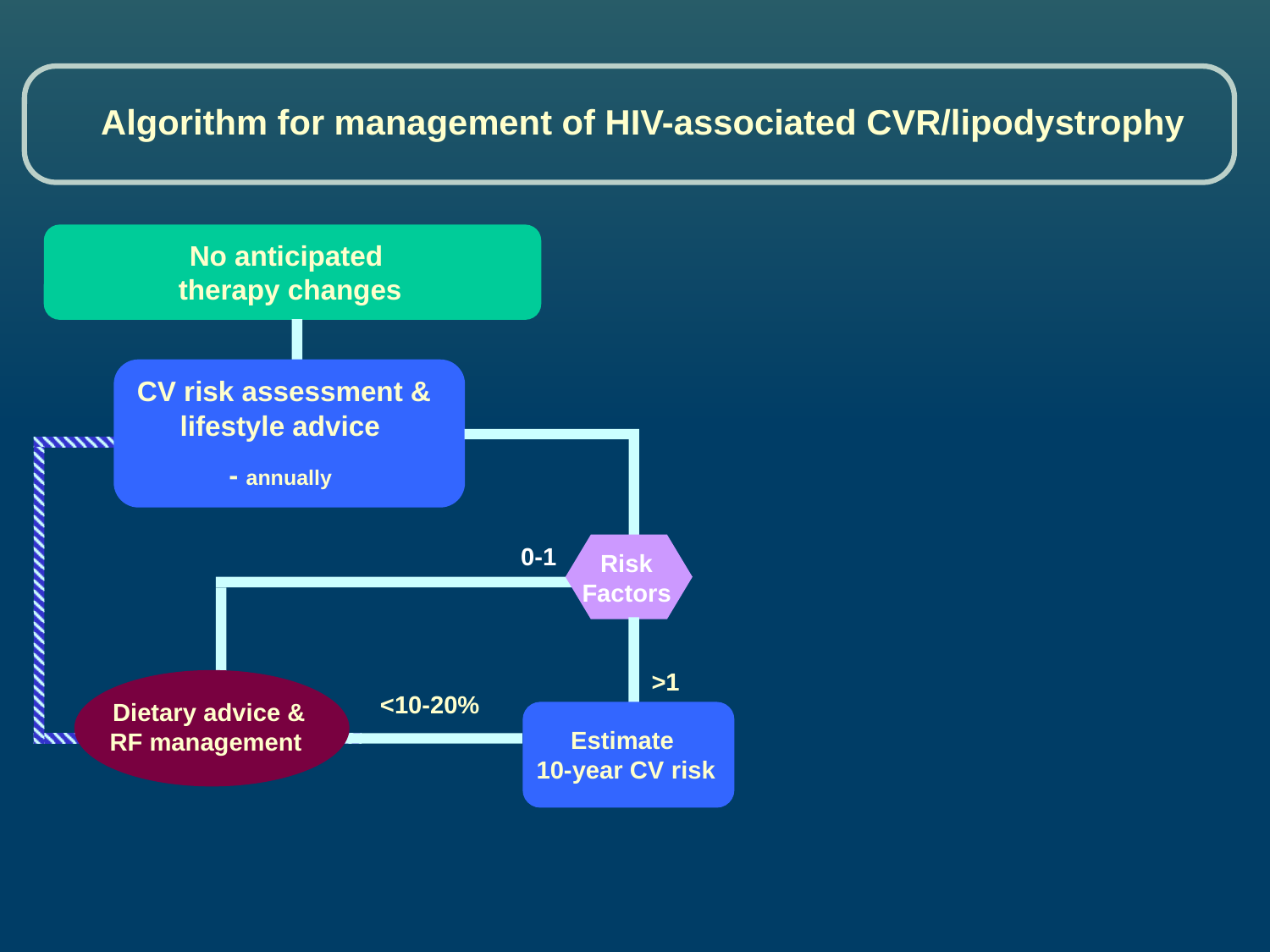

Algorithm for management of HIV-associated CVR/lipodystrophy
No anticipated
therapy changes
CV risk assessment & lifestyle advice
- annually
0-1
Risk Factors
Dietary advice &
RF management
>1
Estimate
10-year CV risk
<10-20%

## Slide 8
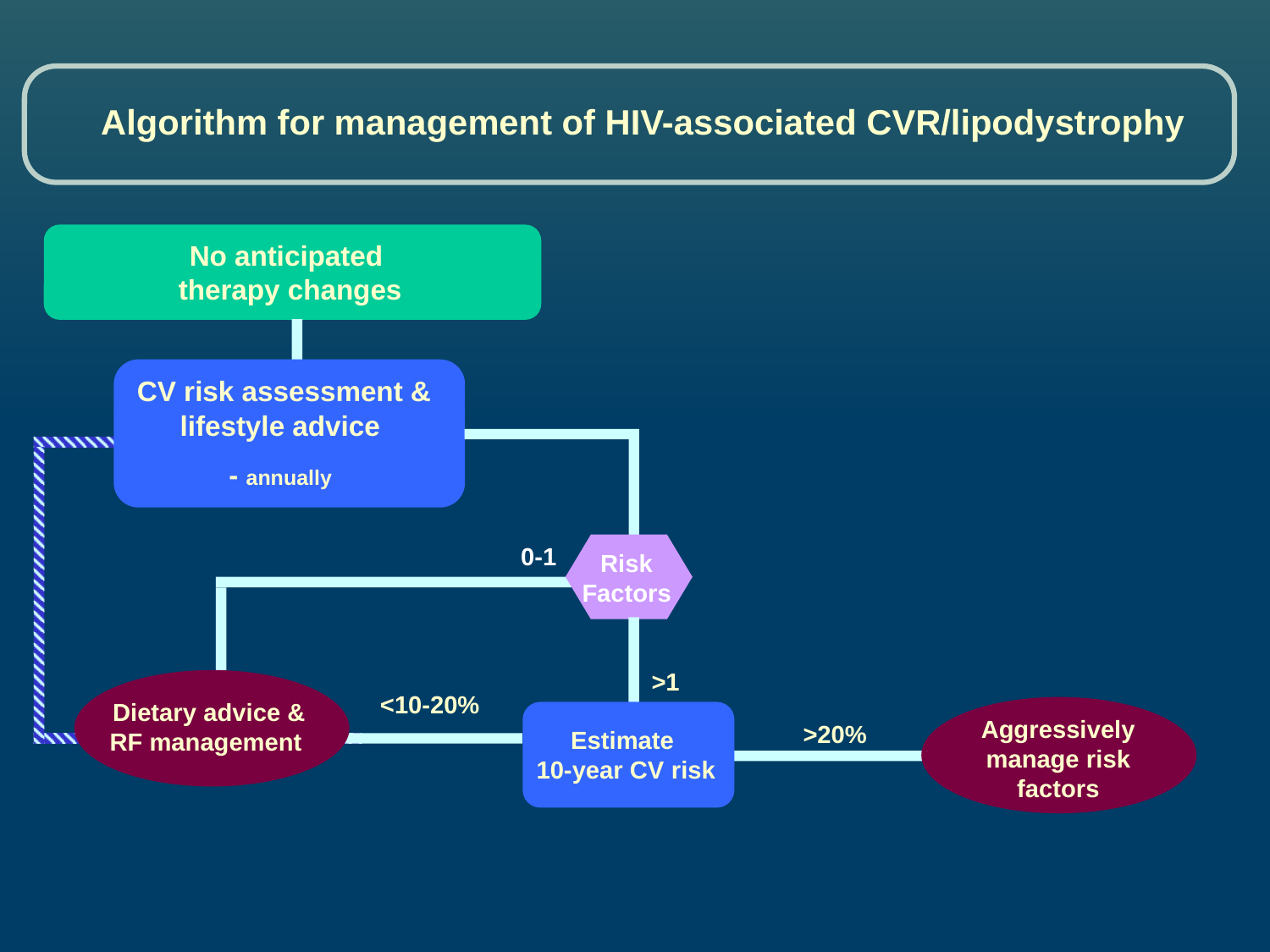

Algorithm for management of HIV-associated CVR/lipodystrophy
No anticipated
therapy changes
CV risk assessment & lifestyle advice
- annually
0-1
Risk Factors
Dietary advice &
RF management
>1
Estimate
10-year CV risk
<10-20%
Aggressively manage risk factors
>20%

## Slide 9
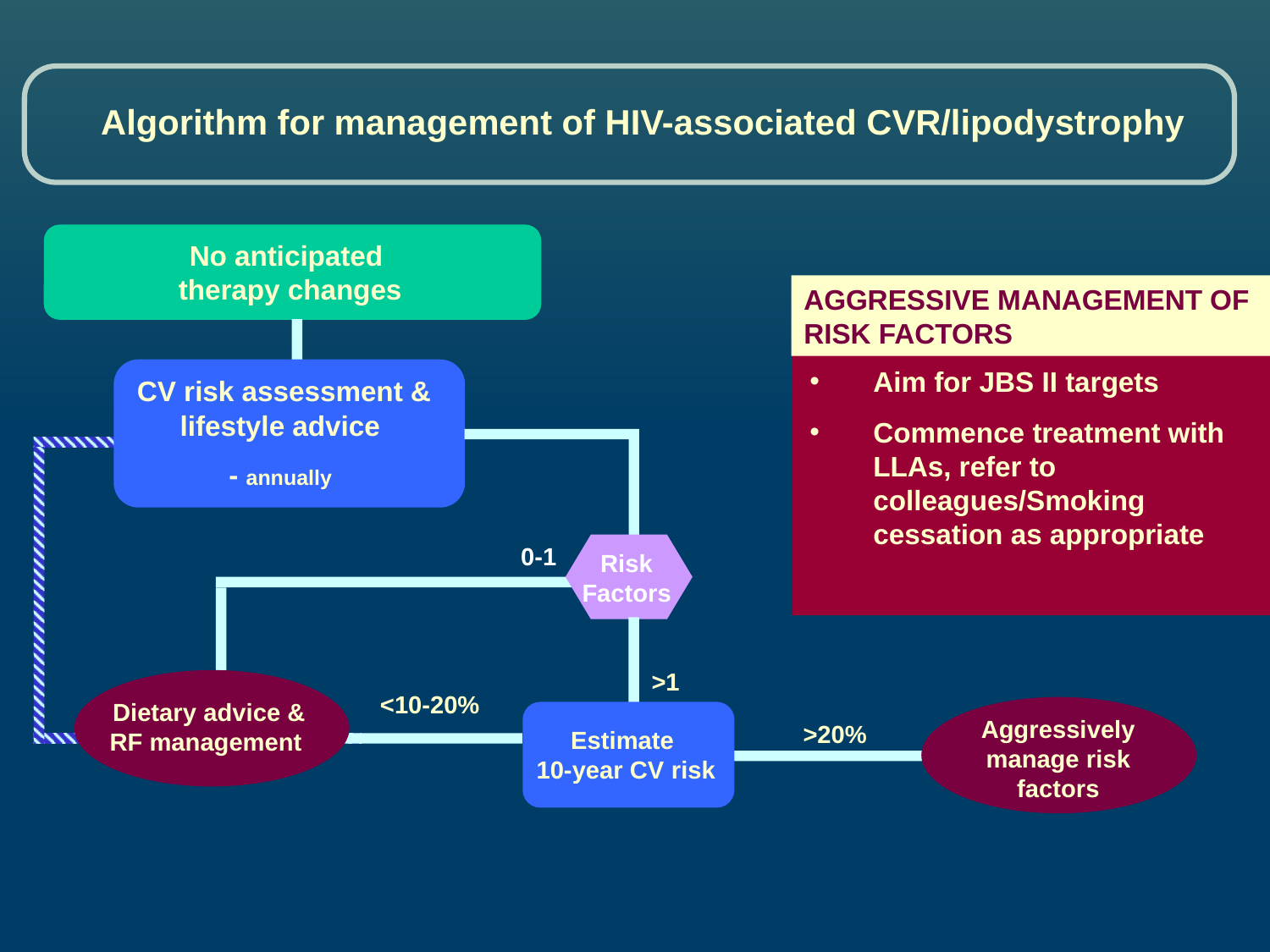

Algorithm for management of HIV-associated CVR/lipodystrophy
No anticipated
therapy changes
AGGRESSIVE MANAGEMENT OF RISK FACTORS
Aim for JBS II targets
Commence treatment with LLAs, refer to colleagues/Smoking cessation as appropriate
CV risk assessment & lifestyle advice
- annually
0-1
Risk Factors
Dietary advice &
RF management
>1
Estimate
10-year CV risk
<10-20%
Aggressively manage risk factors
>20%

## Slide 10
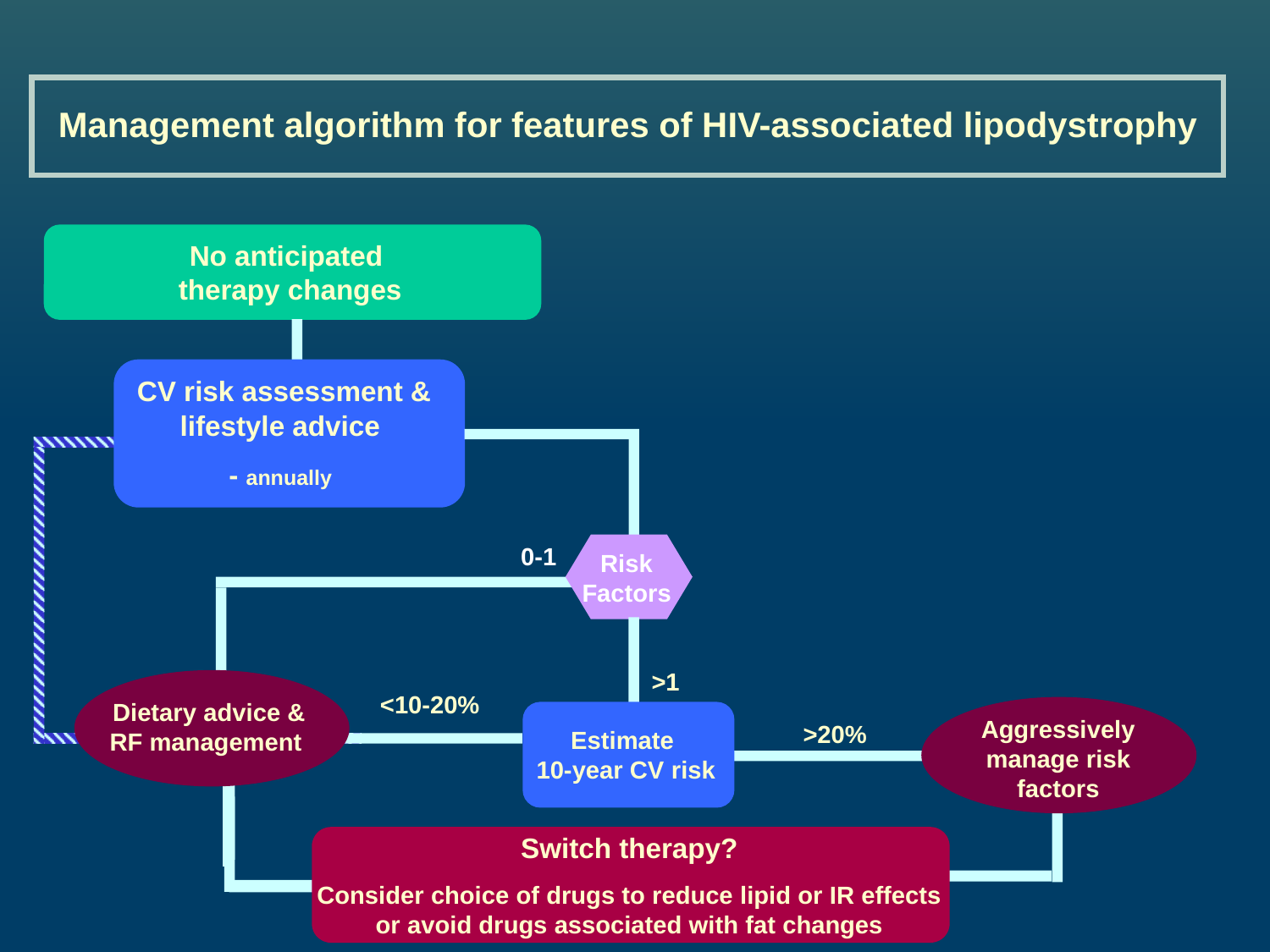

Management algorithm for features of HIV-associated lipodystrophy
No anticipated
therapy changes
CV risk assessment & lifestyle advice
- annually
0-1
Risk Factors
Dietary advice &
RF management
>1
Estimate
10-year CV risk
<10-20%
Aggressively manage risk factors
>20%
Switch therapy?
Consider choice of drugs to reduce lipid or IR effects or avoid drugs associated with fat changes

## Slide 11
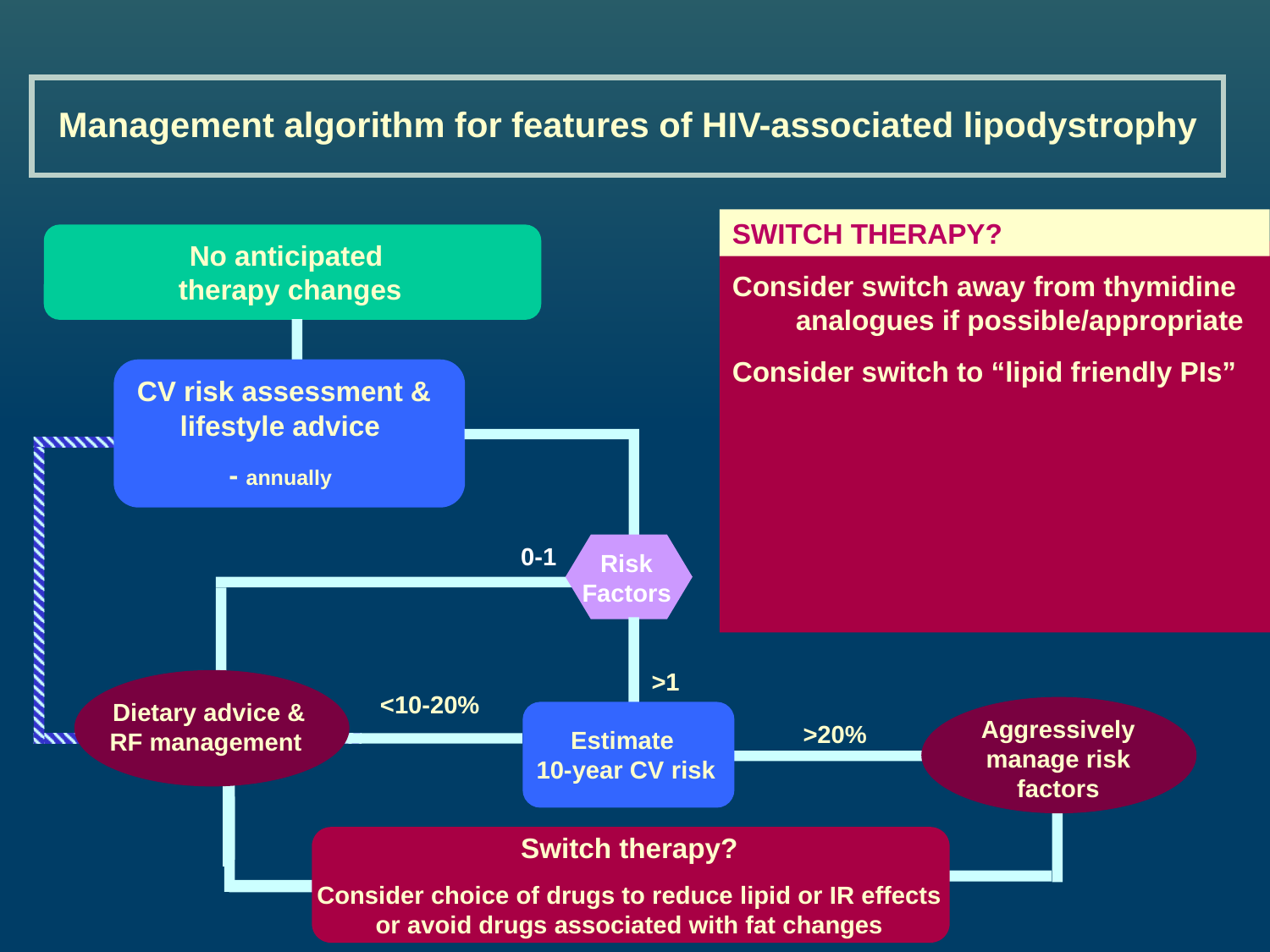

Management algorithm for features of HIV-associated lipodystrophy
SWITCH THERAPY?
No anticipated
therapy changes
Consider switch away from thymidine analogues if possible/appropriate
Consider switch to “lipid friendly PIs”
CV risk assessment & lifestyle advice
- annually
0-1
Risk Factors
Dietary advice &
RF management
>1
Estimate
10-year CV risk
<10-20%
Aggressively manage risk factors
>20%
Switch therapy?
Consider choice of drugs to reduce lipid or IR effects or avoid drugs associated with fat changes

## Slide 12
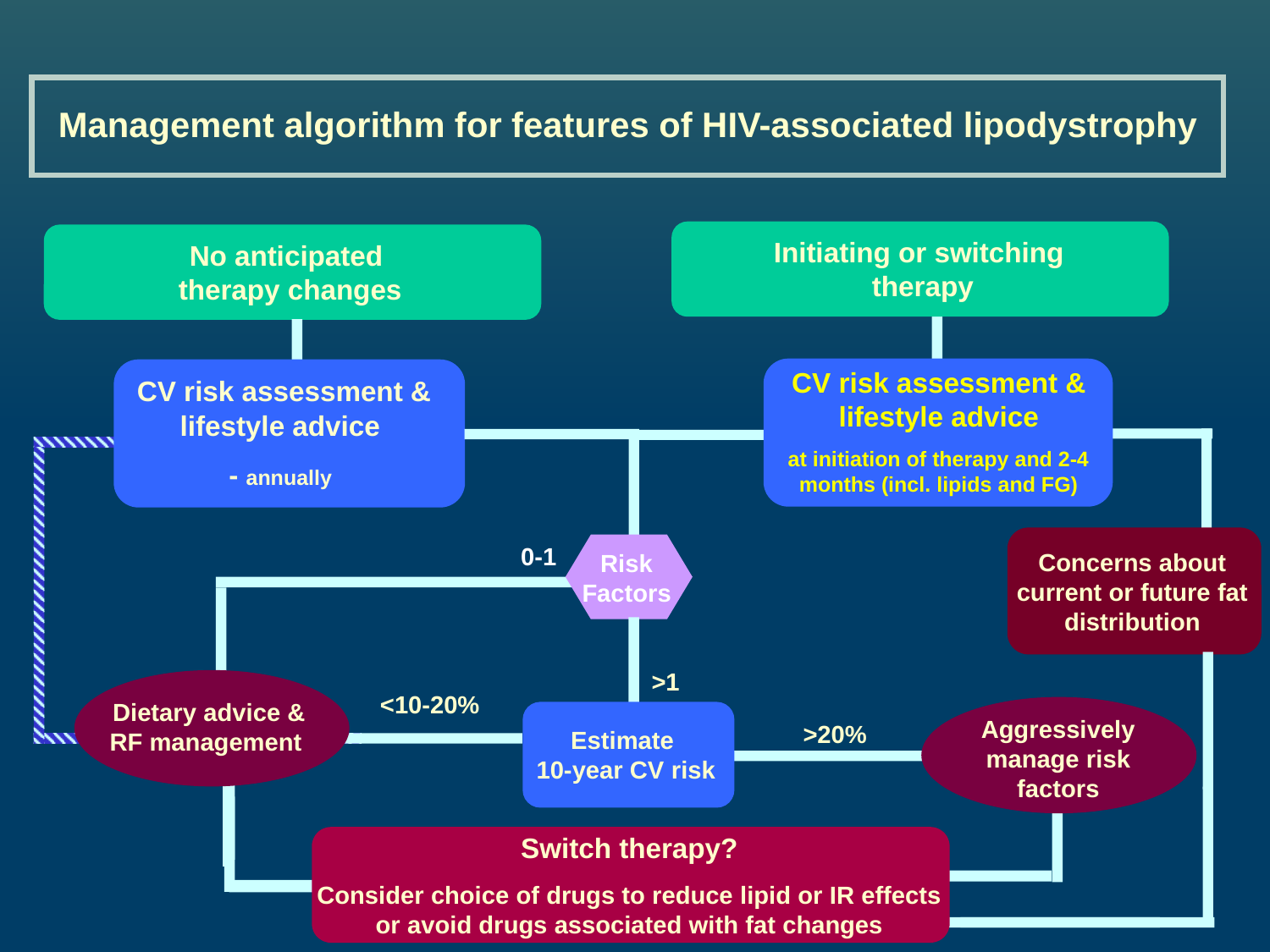

Management algorithm for features of HIV-associated lipodystrophy
Initiating or switching
therapy
No anticipated
therapy changes
CV risk assessment & lifestyle advice
at initiation of therapy and 2-4 months (incl. lipids and FG)
CV risk assessment & lifestyle advice
- annually
0-1
Risk Factors
Dietary advice &
RF management
Concerns about current or future fat distribution
>1
Estimate
10-year CV risk
<10-20%
Aggressively manage risk factors
>20%
Switch therapy?
Consider choice of drugs to reduce lipid or IR effects or avoid drugs associated with fat changes

## Slide 13
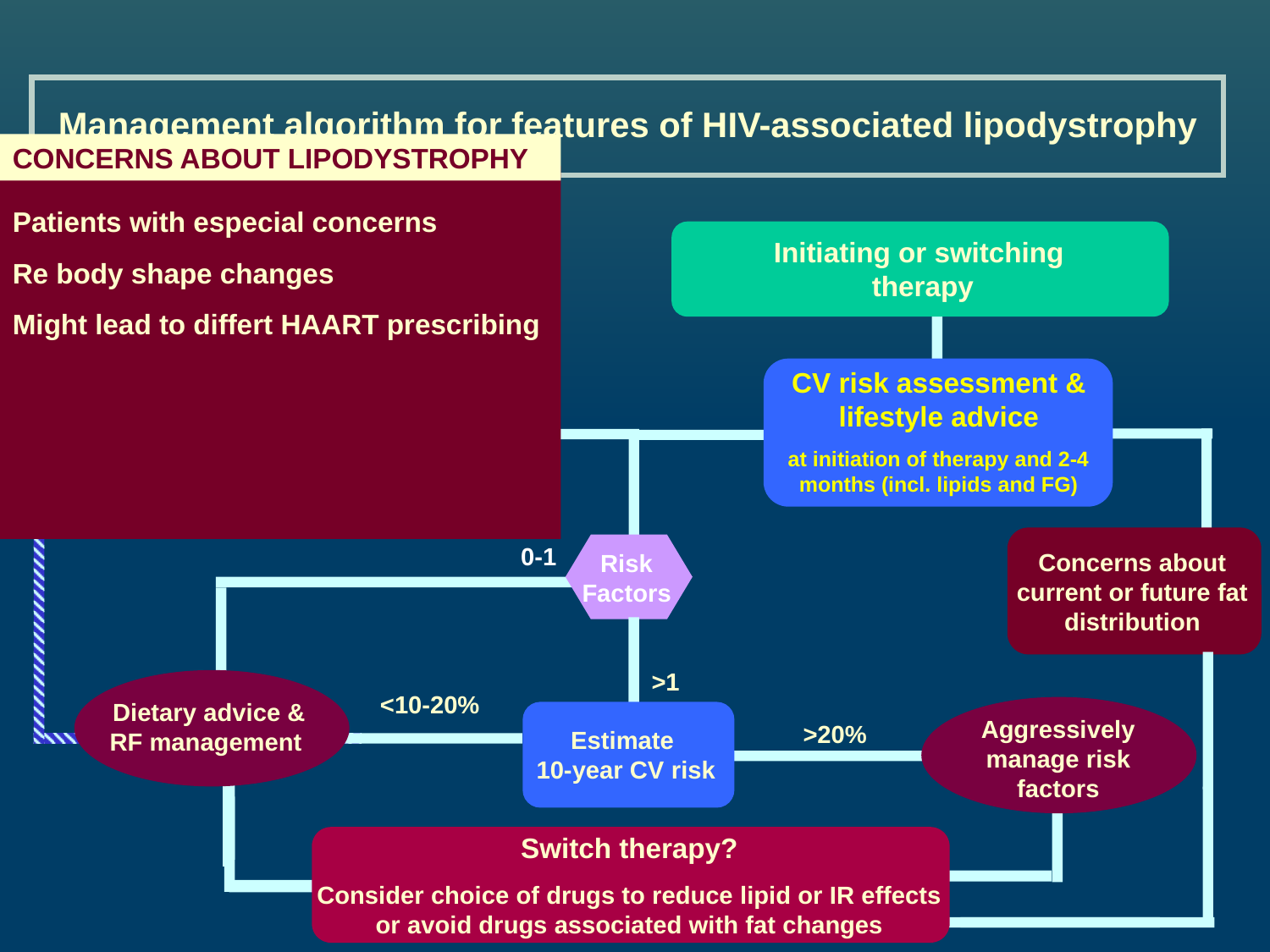

Management algorithm for features of HIV-associated lipodystrophy
CONCERNS ABOUT LIPODYSTROPHY
Patients with especial concerns
Re body shape changes
Might lead to differt HAART prescribing
Initiating or switching
therapy
No anticipated
therapy changes
CV risk assessment & lifestyle advice
at initiation of therapy and 2-4 months (incl. lipids and FG)
CV risk assessment & lifestyle advice
- annually
0-1
Risk Factors
Dietary advice &
RF management
Concerns about current or future fat distribution
>1
Estimate
10-year CV risk
<10-20%
Aggressively manage risk factors
>20%
Switch therapy?
Consider choice of drugs to reduce lipid or IR effects or avoid drugs associated with fat changes

## Slide 14
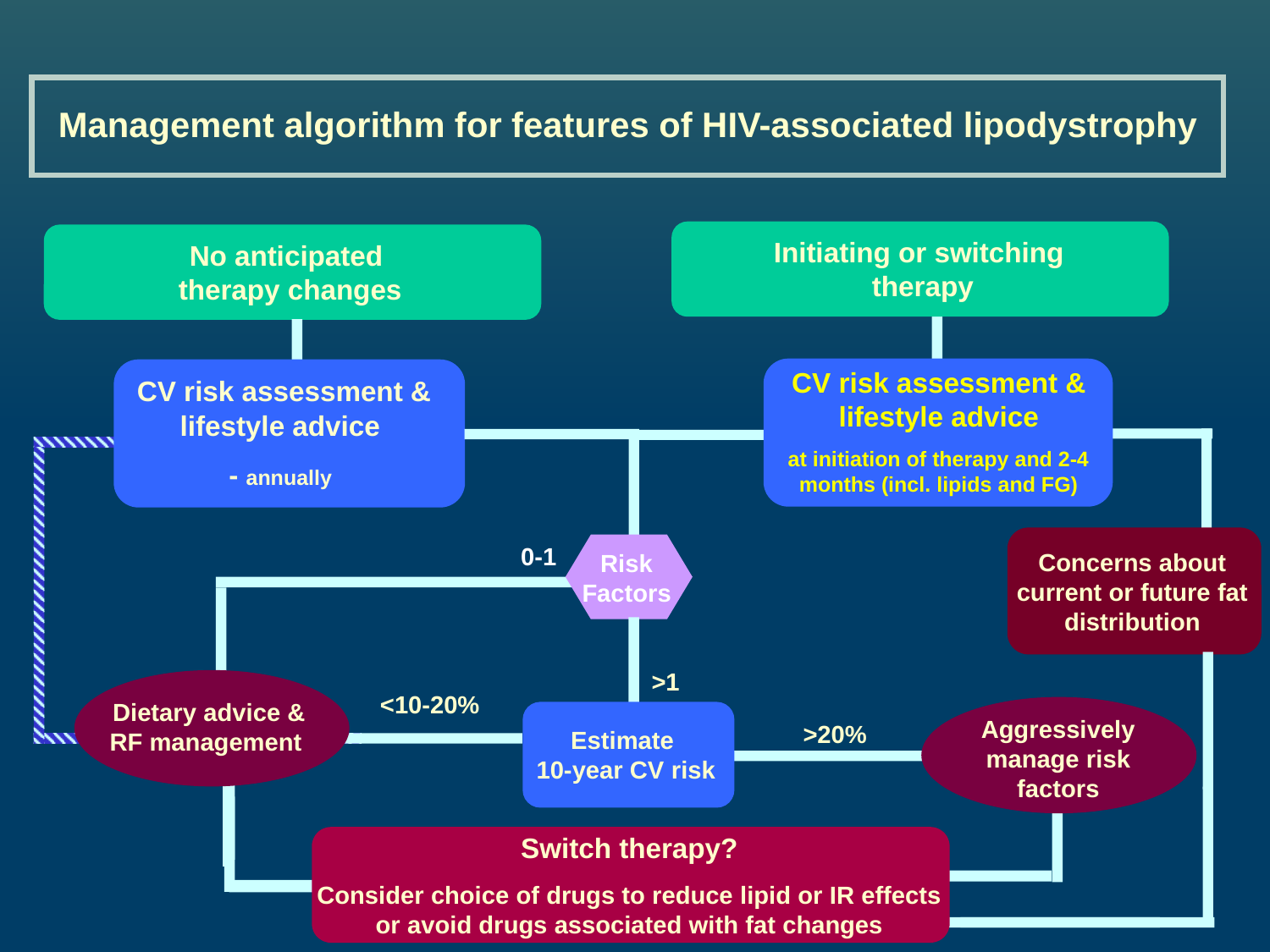

Management algorithm for features of HIV-associated lipodystrophy
Initiating or switching
therapy
No anticipated
therapy changes
CV risk assessment & lifestyle advice
at initiation of therapy and 2-4 months (incl. lipids and FG)
CV risk assessment & lifestyle advice
- annually
0-1
Risk Factors
Dietary advice &
RF management
Concerns about current or future fat distribution
>1
Estimate
10-year CV risk
<10-20%
Aggressively manage risk factors
>20%
Switch therapy?
Consider choice of drugs to reduce lipid or IR effects or avoid drugs associated with fat changes
